# Field assessment of a safe sleep instrument using smartphone technology

**DOI:** 10.1017/cts.2019.446

**Published:** 2019-12-19

**Authors:** Rosemary Nabaweesi, Leanne Whiteside-Mansell, Samantha H. Mullins, Mallikarjuna R. Rettiganti, Mary E. Aitken

**Affiliations:** 1 Pediatrics, College of Medicine, University of Arkansas for Medical Sciences, Little Rock, AR, USA; 2 Arkansas Children’s Research Institute, Little Rock, AR, USA; 3 Family and Preventive Medicine, College of Medicine, University of Arkansas for Medical Sciences, Little Rock, AR, USA; 4 Biostatistics, College of Medicine, University of Arkansas for Medical Sciences, Little Rock, AR, USA

**Keywords:** Smartphone technology, infant safe sleep, photography, health disparity, inter-rater reliability

## Abstract

**Introduction:**

Sudden unexpected infant death is the leading cause of infant mortality with black: white infant mortality remaining at 2:1 for the last decade. Smartphone technology provides a convenient and accessible tool for injury prevention anticipatory guidance among at-risk communities.

**Materials and Methods:**

A convenience sample of pregnant teen mothers who own a smartphone. During a 1-month postnatal home visit, a safe sleep environment survey was administered, infant sleep practices were observed, and mothers trained to take and submit standard infants’ sleep environment photographs. Photographs were independently assessed for inter-rater reliability (IRR) across five sleep safety domains (primary outcome): sleep location, surface, position, presence of soft items, and hazards near the sleep area. Expert and novice coders IRR was measured using Cohen’s kappa coefficient (K). Sleep safety correlation between photographs and observation, and parent report and observation was determined.

**Results:**

Sixteen (57.1%) mothers completed the home visit. Most parents reported infants sleeping supine (78.5) in parents’ bedroom (85.9%). Photographs demonstrated sleep position, soft items without the baby present, and hanging toys had perfect agreement across all three coder pairs. Safe sleep experts’ IRR demonstrated perfect agreement for sleep location, position, and soft items. While 83.8% of parents were observed putting their infants down to sleep on their back, 78.5% of parents reported doing the same and 82.4% of the photographs demonstrated supine infant sleep position.

**Conclusion:**

Using photographs, coders can reliably categorize some key infant sleep safety aspects, and photograph sleep safety is comparable to parent report and direct observation.

## Introduction

The National Center for Health Statistics and Center for Disease Control and Prevention report that, in the USA, nearly 4000 infants die annually from sleep-related causes [1, 2] including sudden unexpected infant death (SUID) and sudden infant death syndrome (SIDS). SUID is the leading cause of infant mortality in the USA among children aged 1 month to 1 year [1, 3]. SUID is defined as the sudden and unexpected death of an infant without obvious cause before an investigation [3–5]. After a full investigation, SUID may be classified as SIDS, suffocation, trauma, metabolic disease, or unknown. SIDS is defined as sudden, unexpected infant death that cannot be explained after completion of a scene investigation, autopsy, and clinical history review [6].

Sleep-related deaths have plateaued since the initial decline following the 1992 Back-to-Sleep campaign that was disseminated by the National Institutes of Child Health and Human Development (NICHD) and the American Academy of Pediatrics (AAP) [4–6]. Subsequently, the AAP and NICHD developed evidence-based safe sleep recommendations [7] to educate parents, caregivers, and health care providers [7, 8]. The recommendations’ key messages are four-fold: (1) keep infants on their back for all sleep, including nighttime and naps; (2) use a firm sleep surface such as a mattress in a safety-approved crib; (3) keep soft bedding and items such as bumper pads, blankets, and stuffed animals out of infants’ sleep area [2]; and (4) have the infant share the parents’ room but not the parents’ bed.

The difficulty in implementing these recommendations across all communities is illustrated by the disparities in infant death rates among races and between rural and urban areas [9]. Black infants remain twice as likely to die from SUID as White infants [9, 10]. Similarly, the number of rural infant sleep-related deaths is four times the number of urban infant sleep-related deaths [10]. White parents (84%) place infants on their back to sleep more often than Black parents (62%) [11, 12]. Additionally, Black parents are twice as likely to share an adult bed with their infants compared to White parents [13, 14].

Studying, promoting, and delivering these sleep recommendations to reduce SUID require a targeted approach. One challenge is assessing an infant’s risk for SUID when resources are limited. Direct observation of the sleep environment is considered the gold standard for assessing infants at risk and for promoting compliance with injury-preventing recommendations [15, 16]. For example, home-visiting educators are trained to assess infant sleep environments and provide education to increase protective factors such as breastfeeding [17, 18]. However, due to families’ limited resources, many infant homes do not qualify for home-visiting programs, or parents are reluctant to participate because of the invasive nature of home visits [17, 19]. An alternative to direct observation is parental reporting of safe sleep practices. The *Newborn Sleep Safety Survey*, developed and described by the study investigators [20], is a validated instrument to assess infant risk and intervention success. To build upon this work, a cost-effective safe sleep assessment methodology using smartphone photography was developed.

Using photographs to assess an infant’s sleep environment could potentially identify key safety issues related to the infant’s sleep location, space, position, and items within and near the sleep space. The premise is that smartphone photography will be as effective as having a home visitor assess the sleep environment or parent report sleep practices using the *Newborn Sleep Safety Survey* [20] (see Supplement).

This feasibility study aimed to develop and standardize a method for parents to take and transmit photographs of the infant’s sleep environment using a smartphone. A secondary aim was to test the usefulness of the photographs by evaluating the ability of safe sleep expert and novice coders to implement the coding protocol and thus determine the infant’s sleep risk. Investigators examined agreement between two safe sleep expert coders: an expert and a novice safe sleep coder, and an expert coder and expert home visitor. Also examined was the correlation between sleep safety assessments determined by photographs and direct observation, as well as by parental reports and direct observation.

## Materials and Methods

### Study Design and Setting

This study was conducted as part of an ongoing randomized clinical trial. Parent study aim was to determine the effectiveness of a prenatal safe sleep educational intervention on intergenerational safe sleep knowledge and skills for teen mothers (TMs). A convenience sample consisting of new or expectant TM was recruited from the Supplemental Nutritional Program for Women, Infants, and Children, obstetrics, and pediatric outpatient clinics. A 90-minute home visit was scheduled for approximately 1 month after birth, or as soon as possible, for new mothers. An experienced research assistant conducted the home visit, which included a parent interview, sleep environment observation, and photographic documentation training. Mothers also received supportive feedback on their safe sleep practices and their infant’s sleep environment, including referrals to hospitals and other community resources to obtain safe sleep equipment, such as portable play yards. Parents were asked to text photos 2–4 weeks after the home visit and received two $25 gift cards for the home visit and for sending photographs. Study data were collected and managed using Research Electronic Data Capture (REDCap) tools hosted at our institution [21]. REDCap is a secure, web-based application designed to support data capture for research studies, providing an intuitive interface for validated data entry, audit trails for tracking data manipulation and export procedures, automated export procedures for seamless data downloads to common statistical packages, and procedures for importing data from external sources. The institution’s review board expedited approval of all study activities.

### Study Population, Screening, and Enrollment

Teenage mothers in the third trimester of pregnancy or up to 2 months post-delivery were recruited. Eligibility screening included the following inclusion criteria: mother (1) is between 13 and 19 years old and in the last trimester or up to 2 months post-delivery; (2) owns a smartphone that can take and send photographs; (3) will live within designated geographic area for at least 4 months after delivery to facilitate home visits; and (4) speaks English. Teenagers whose head of household declined permission to access the home were excluded from the study. Recruitment strategies included flyers, information sheets, word of mouth, social media, and key informants at safe sleep partner organizations.

### Study Procedures, Measures, and Data Collection

#### Sleep environment parent report

The *Newborn Sleep Safety Survey* [20] was designed by study investigators to assess the family’s living arrangements and the infant sleep environment. The survey assessed six key AAP recommendations including (1) supine sleep position, (2) firm sleep surface, (3) separate sleep location but in same room, (4) no soft items or loose bedding in sleep area, (5) not an overly warm sleep environment, and (6) pacifier use. The survey employed a Likert scale for questions referring to frequency of safe sleep practices. The options included “every time,” “more than half the time,” “about half the time,” “less than half the time,” “never,” and “don’t know.” Results demonstrated acceptable agreement between parental reports and observation, with one item showing strong agreement and four items showing moderate agreement [20].

#### Sleep environment observation

An observation assessment that was developed to closely match the survey items was used. This assessment was designed to document the actual infant sleep environment and parents’ demonstrated sleep practices, including sleep location, surface, position, and other recommendations identical to the survey. A standard checklist was completed during the home visit.

#### Sleep environment photography protocol and parent training

The research assistant took sample photographs with their smartphone and demonstrated to TM. TM practiced taking the six photographs with their own smartphone. The research assistant verified that TM was taking photographs correctly. The research assistant asked TM to send the training photographs to study phone to verify compatibility. The photographs were downloaded from the study phone and uploaded into a REDCap database. Researchers developed, piloted, and standardized a photography protocol to document infants’ sleep environment using six photographs taken from three vantage points. The protocol included two photographs of the entire room taken from opposite ends of the infant’s sleep location; two photographs from the side of the crib, with and without a doll or the infant; and two photographs taken from above the sleep area with and without a doll or the infant. The photo coding protocol assessed the following: (1) type of sleep space (crib, bassinette, and adult bed), (2) room versus bed sharing (location), (3) sleep position, (4) presence of bumper pads, loose bedding, or soft items, and (5) hazards near the sleep area. Safe sleep experts coded the training photographs and follow up photographs using the photo coding protocol in the REDCap database.

#### Assessment

The primary outcome was sleep safety inter-rater reliability (IRR) of coders examining photographs. IRR measures the extent to which coders assign the same sleep safety measure using the same data source. Two types of safe sleep coders were used: (1) experts who were injury prevention specialists with more than 2 years’ experience conducting infant safe sleep education and research, and (2) novices who were injury prevention specialists with no experience conducting safe sleep research or education but who had experience in other unintentional injury prevention areas. To assign sleep safety scores across the five domains, novice coders used a coding guide that provided detailed descriptions of each domain selection.

Secondary outcomes were correlation between sleep safety assessments via researcher observation and photography methodology, assessment correlation between parental reports and observation, and parental behavior change following feedback on observed sleep practices during the home visit. The hypotheses were that the IRR among photography content coders would be strong, and that both the correlation between sleep safety using observation and photographs and sleep safety from the parental report and observation would be high. Reliability for each item was estimated using a Cohen’s kappa statistic and reported a one-sided 95% confidence interval. A kappa (K) agreement of 0.6 or higher was considered an indication of high correlation, based on McHugh’s K statistic interpretation [22].

## Results

### Study Participant Characteristics

**Fig. 1.**
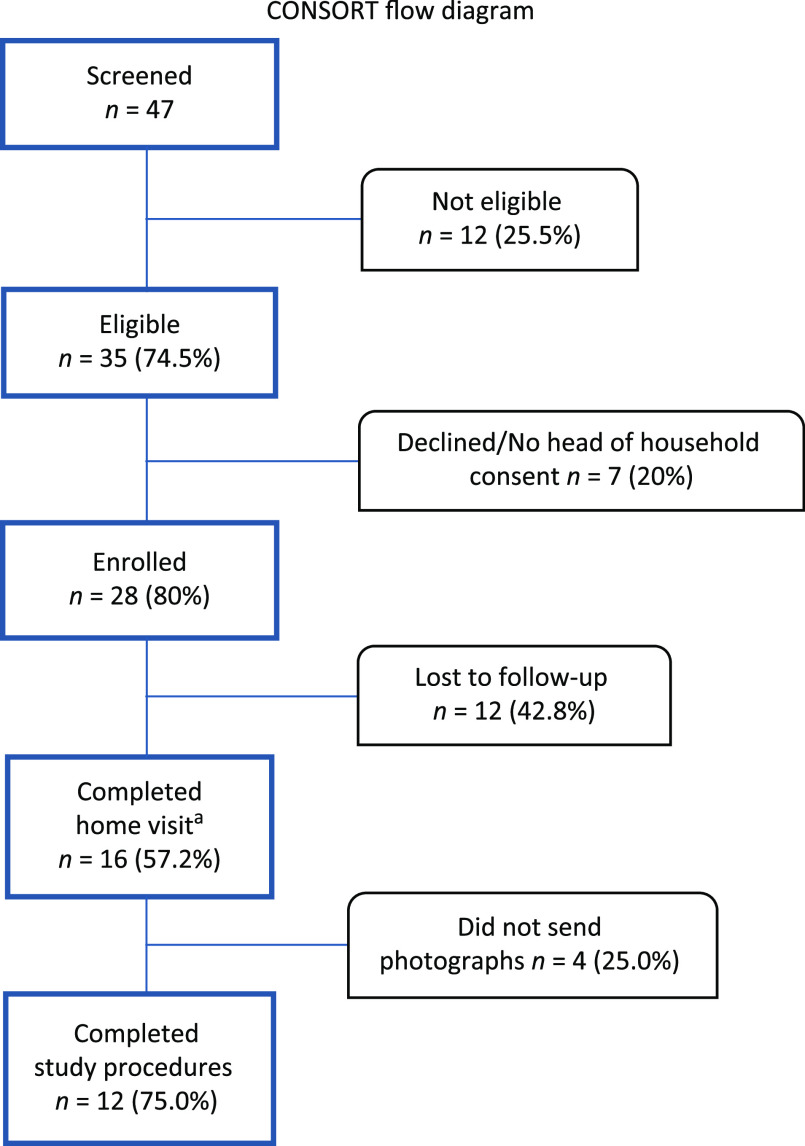
Participant eligibility, enrollment, and follow-up. ^a^Health educator observation and parent report (using survey) of the infant’s sleep environment conducted during the home visit.

Forty-seven participants were screened for eligibility and 35 (74.5%) met eligibility criteria (Fig. 1). Eighty percent (*n* = 28) of eligible participants were enrolled. Sixteen mothers completed the home visit and 12 completed all study procedures. Sixty-four percent (*n* = 18) were Black and 21% (*n* = 6) were White, with a median age of 18 years (interquartile range, 2.26 years). Twelve mothers (42.8%) sent usable data. Four mothers (25%) did not send photographs because their phone service was disconnected.

### Photograph Inter-Rater Reliability (IRR)

Based on the photographs, all of the coding pairs, regardless of safe sleep expertise, agreed on assessments of sleep position, soft items without the baby present, and hanging toys (Table 1). Conversely, there was no agreement between any coding pairs in identifying hazards such as electric cords near sleep area, *K* = 0.00. Some coders identified hazards while others did not. The IRR between safe sleep experts demonstrated perfect agreement for sleep location, sleep position, and soft items in sleep area without baby present (*K* = 1.00) and was strong for sleep surface (*K* = 0.70) (Table 1). The IRR between expert coders for items in the sleep area (with the baby present) varied at *K* = 0.00, *K* = 0.46, and *K* = 0.59 for soft items, no items, and loose bedding, respectively.

**Table 1.** Sleep safety inter-rater reliability using photographs

	Inter-rater reliability kappa (*K*)		
Sleep domain	Both coders SSE	Novice^a^ and SSE coder	SSE and home visitor coder
Room location (parents, nursery)	1.00	0.33	1.00
Surface (crib, bassinet, play yard)	0.70	0.62	0.78
Position	1.00	1.00	1.00
Items in sleep area without baby present			
Loose beddings	1.00	0.20	1.00
Soft items (stuffed animals)	1.00	1.00	1.00
None	–	–	–
Items in sleep area with baby present			
Loose bedding	0.59	0.78	0.59
Soft items (stuffed animals)	0.00	0.00	0.00
None	0.46	0.10	0.46
Hazards near sleep area			
Electric/blinds cords/fans	0.00	0.00	0.00
None	0.16	0.52	0.16
Hanging toys	1.00	1.00	1.00

SSE, safe sleep experts.

a Used a coding guide.

The IRR between the novice and expert for identifying no hazards near the sleep area was higher (*K* = 0.52) than the IRR between safe sleep experts (*K* = 0.16). The IRR between expert and novice coders was lower for sleep location (*K* = 0.33) and sleep surface (*K* = 0.62). However, like the safe sleep expert coding pairs, sleep position, soft items in actual sleep area without baby, and hanging toys had perfect agreement (*K* = 1.00). The IRR agreement between coders with varying safe sleep knowledge for soft bedding without baby present was low (*K* = 0.20) but higher with baby present (*K* = 0.78). The IRR for no hazards observed near actual sleep area increased to *K* = 0.52. The IRR between home visitor and expert coder for all domains except for one (sleep surface) was identical to IRR between expert coders.

### Sleep Safety Using Photographs and Parental Reports Compared to Home Visitor Observation

Sleep safety determined by parental reports and training photography were compared to home visitor observation, the ideal method for assessing sleep safety. Table 2 shows the percentage of safe sleep practices observed, mothers who reported safe sleep practices “most of the time,” and the safe sleep environment illustrated on the photographs. Also reported were the Kappa agreement of sleep safety determined by parental reports and observation, and agreement between sleep safety assessments determined by photographs and observation (see Table 3 for interpretation of Kappa scores).

**Table 2.** Comparison of safe sleep practices as obtained from parent report and photographs to the gold standard of observation

Safe sleep domain	Safe behavior/environment (*n* = 17)	Observed behavior^a^	Parent report		Photographs of sleep environment	
		% Safe	% Safe^b^	Agree^c^	% Safe	Agree^c^
Sleep position	Back	76.5	76.5	0.89*	82.4	0.81*
Sleep location	Parent’s room	94.1	82.4	0.84*	82.4	0.84*
Bedding safety	No pillows	88.2	88.2	0.88*	82.4	0.77*
	No blankets/sheepskin in the sleep area	41.2	70.6	0.17*	58.8	0.43
Soft items	No stuffed animals in the sleep area with your baby	82.4	76.5	0.83*	94.1	0.45*
Hazards near sleep area	No hazards close to sleep area that baby could touch	70.6	88.2	0.48*	41.2	0.23
Sleep surface	Crib, pack and play or bassinette	82.3	76.4	0.82*	82.3	1.00*

* Kappa agreement was statistically significant at the 0.05 significance level.

a Simulation with a doll.

b Percent behavior is observed/reported or identified on photograph for “Most of the Time” on Parent report.

c Cohen’s kappa inter-rater reliability coefficient.

**Table 3.** Interpretation of Kappa Scores [23]

Kappa	Agreement
<0	Less than chance agreement
0.01–0.20	Slight agreement
0.21–0.40	Fair agreement
0.41–0.60	Moderate agreement
0.61–0.80	Substantial agreement
0.81–0.99	Almost perfect agreement

Most parents (76.5%) were observed putting their infants down to sleep on their back, 76.5% reported doing so, and 82.4% of the photographs demonstrated a supine (back) infant sleep position. The agreement between parental reports and observed practices was strong (*K* = 0.85), as was agreement between the photographs and observation (*K* = 0.89). The research assistant observed that 94.1% of infants slept in the parents’ room. Similar to parent reporting, 82.4% of photographs showed that the infant slept in the parents’ room. Although sleep safety agreement between parental reports (82.4%) and observation (94.1%) for sleep location was excellent (*K* = 0.84), agreement between the photographs and observation was perfect (82.4%). Results suggest that smartphone photograph use in sleep safety assessment may be comparable to observation across infant sleep domains of location, position, and surface.

### Comparing Infant Sleep Environments at Training and 1-Month Photographs

Lastly, sleep domains illustrated by training photographs were compared to domains illustrated by photographs sent 1 month after the home visit. Ten out of 12 (83%) mothers illustrated increased adherence to safe sleep recommendations across the five domains.

## Discussion

Direct observation by safety-trained research staff is considered a more objective means of assessing uptake of a behavioral intervention than subject report alone to avoid response bias. To assess infant sleep safety practices using direct observation, home visits may be required, which may be difficult due to cost, shortage of personnel, and lack of time resources. In addition to being costly, the intrusive nature of home visits may limit subject participation. While direct observation introduces the Hawthorne effect, parental reports are susceptible to social desirability bias.

In response to this problem, the investigators developed a standardized and cost-effective method to assess infant sleep safety using photographs. The results of this study demonstrate that TMs could take usable photographs of their infants’ sleep environment and potentially send those photographs to a study cell phone via text message. The results also suggest that both safe sleep injury prevention experts and novices can assess sleep safety using photographs for domains of sleep position, sleep surface, and items in the sleep area without baby present. Reliably assessing subjective sleep domains, including items in the sleep area with baby present and hazards near the sleep area, was challenging for all coders. Additionally, novice coders were not able to reliably assess sleep location from the photographs.

Sleep safety correlation between training photographs and observation was high for location, position, surface, and hazard domains. This correlation was comparable to sleep safety correlation between parental reports and observation for sleep location, position, and hazards. The 1-month post-home-visit photographs illustrated that most parents adopted the safe sleep recommendations provided at the home visit.

Using photographs to demonstrate compliance with safe sleep and possibly other safety recommendations could provide a cost-effective assessment and evaluation tool with both cost and time-saving advantages over direct observation through home visits. The potential for direct feedback concerning safety issues demonstrated in the photos may support safe sleep interventions in clinical and community settings. Once the effectiveness and feasibility of smartphone photography is fully established, potential increases in safe sleep practices may be realized. The work presented here aligns with that of Herring *et al.* [24], who used a randomized controlled trial to demonstrate the acceptability, feasibility, and adaptability of a smartphone technology-based post-partum weight loss intervention. Their study comprised empirically supported behavior-changing strategies, daily skills, and self-monitoring text messages with personalized feedback, among 18- to 19-year-old mothers [24]. Likewise, using a survey, both Swindle *et al.* and Omaki *et al.* demonstrated that low-income parents of young children had daily usage rates of the internet, texting, and cell phone of greater than 60% [25, 26]. Cell phone observation may provide an acceptable alternative to direct observation for other injury prevention mechanisms such as appropriate seatbelt placement, helmet or lifejacket fit, and smoke detector installation.

Approximately 42.8% of eligible mothers successfully sent usable photographs, suggesting feasibility of our approach. However, one weakness of this study was loss to follow-up. This was a feasibility study conducted as part of a clinical trial. The loss to follow-up in the feasibility study was mirrored in the parent study, a randomized clinical trial. We are in the process of developing a larger independent study proposal using lessons learned from the feasibility study. Reducing the time lag between initial screening, recruitment, scheduled home visit, and prompt for photographs may curb this loss, but a challenge that needs to be addressed in a larger follow-up study is maintaining connection to participants despite phone service interruptions. The second weakness was that the focus on TMs may limit generalizability to the wider parent population and parents with less access to smartphones. Further study with a larger and more broadly representative sample of parents is warranted. Finally, the lack of reliable risk assessments may be addressed with adjustments to the photography protocol such as using video or changing vantage points from which photographs are taken.

## Conclusion

Smartphone photography is a promising assessment tool for health information. Safe sleep injury prevention experts and novices may reliably categorize some key safety aspects of infant sleep practices using smartphone photographs, and the safety assessment from photographs correlates well with direct observations of the sleep environment. However, further study is needed to fully characterize sleep environments using photographs alone.

## Supplementary material

For supplementary material accompanying this paper visit https://doi.org/10.1017/cts.2019.446. click here to view supplementary material
