# Supplementary material for: Field assessment of a safe sleep instrument using smartphone technology
Source: J Clin Transl Sci. 2019 Dec 19;4(5):451–6. doi: 10.1017/cts.2019.446 (PMC7681145; doi:10.1017/cts.2019.446)
Supplement: Supplementary file 1 [file S2059866119004461sup001.docx]

| **AAP Safe Sleep Recommendation** | **Survey Questions** |
| --- | --- |
|  |  |
| Sleep position | 1. How often do you put your baby down to sleep on his/her back? |
| Sleep surface | 1. The mattress in the (CRIB) is firm? |
| Sleep location | 1. At night, the baby sleeps in the same room as you or another adult? |
|  | 1. How often do you and your baby sleep in the same bed? With someone else? |
|  | 1. How often do you put your baby down to sleep in an adult bed? |
|  | 1. Where do you put your baby down most? (response of adult bed) |
| Bedding safety — soft or loose bedding | 1. When your baby is sleeping, how often is there a pillow in the (CRIB) for him/her to rest his/her head on? |
|  | 1. When your baby is sleeping, how often is there a bumper pad around the edges of the (CRIB)? |
|  | 1. How often is there a cushion, pillow, heavy blanket, or sheepskin, on top of the mattress but underneath the baby? |
|  | 1. How often is there a stuffed animal in the (CRIB) with your baby? |
|  | 1. When your baby is sleeping, how often does the (CRIB) have a mattress in it — one that fills up the whole bottom of the (CRIB) and doesn't leave any space between the mattress and the edges of the (CRIB)? |
|  | 1. How often do you use a wedge or something else to hold the baby in a particular position while s/he sleeps, or to keep him/her from rolling? |
| Overheating | 1. How often is there a heavy blanket, comforter, or bedspread in the (CRIB) with your baby? |
| Offer a pacifier at nap time and bedtime | 1. How often do you give your baby a pacifier when you put him/her down for sleep? |

**Supplement 1: Newborn Sleep Safety Survey**

**Source:** Whiteside-Mansell L, Nabaweesi R, Caballero AR, Mullins SH, Miller BK, Aitken ME. Assessment of Safe Sleep: Validation of the Parent Newborn Sleep Safety Survey. *J Pediatr Nurs* 2017; 35:30-35
